# Ultra-High Strength and Specific Strength in Ti_61_Al_16_Cr_10_Nb_8_V_5_ Multi-Principal Element Alloy: Quasi-Static and Dynamic Deformation and Fracture Mechanisms

**DOI:** 10.3390/ma18143245

**Published:** 2025-07-10

**Authors:** Yang-Yu He, Zhao-Hui Zhang, Yi-Fan Liu, Yi-Chen Cheng, Xiao-Tong Jia, Qiang Wang, Jin-Zhao Zhou, Xing-Wang Cheng

**Affiliations:** 1 School of Materials Sciences and Engineering, Beijing Institute of Technology, Beijing 100081, China; 3120215537@bit.edu.cn (Y.-Y.H.); liuyifan1208@sina.com (Y.-F.L.); cheng_yichen@outlook.com (Y.-C.C.); 15732028671@163.com (X.-T.J.); 3120225558@bit.edu.cn (Q.W.); 18256194391@163.com (J.-Z.Z.); 2 Tangshan Key Laboratory of High-Performance Metals and Ceramics, Tangshan Research Institute of Beijing Institute of Technology (BIT), Tangshan 063000, China

**Keywords:** ultra-high specific strength, strain rate sensitivity, dislocation cross-slip, rotational dynamic recrystallization, forced shear deformation, multi principal element alloy

## Abstract

This study investigates the deformation and fracture mechanisms of a Ti_61_Al_16_Cr_10_Nb_8_V_5_ multi-principal element alloy (Ti61V5 alloy) under quasi-static and dynamic compression. The alloy comprises an equiaxed BCC matrix (~35 μm) with uniformly dispersed nano-sized B2 precipitates and a ~3.5% HCP phase along grain boundaries, exhibiting a density of 4.82 g/cm^3^, an ultimate tensile strength of 1260 MPa, 12.8% elongation, and a specific strength of 262 MPa·cm^3^/g. The Ti61V5 alloy exhibits a pronounced strain-rate-strengthening effect, with a strain rate sensitivity coefficient (m) of ~0.0088 at 0.001–10/s. Deformation activates abundant {011} and {112} slip bands in the BCC matrix, whose interactions generate jogs, dislocation dipoles, and loops, evolving into high-density forest dislocations and promoting screw-dominated mixed dislocations. The B2 phase strengthens the alloy via dislocation shearing, forming dislocation arrays, while the HCP phase enhances strength through a dislocation bypass mechanism. At higher strain rates (960–5020/s), m increases to ~0.0985. Besides {011} and {112}, the BCC matrix activates high-index slip planes {123}. Intensified slip band interactions generate dense jogs and forest dislocations, while planar dislocations combined with edge dislocation climb enable obstacle bypassing, increasing the fraction of edge-dominated mixed dislocations. The Ti61V5 alloy shows low sensitivity to adiabatic shear localization. Under forced shear, plastic-flow shear bands form first, followed by recrystallized shear bands formed through a rotational dynamic recrystallization mechanism. Microcracks initiate throughout the shear bands; during inward propagation, they may terminate upon encountering matrix microvoids or deflect and continue when linking with internal microcracks.

## 1. Introduction

Multi-principal element alloys (MPEAs) are a novel class of alloys characterized by the presence of four or more principal elements [1,2], each with a molar fraction exceeding 5%. Compared to traditional alloys, MPEAs exhibit four core effects [3,4], enabling them to surpass conventional alloys in certain performance aspects [2,4], thus becoming a recent research hotspot. MPEAs can be classified in various ways. For instance, based on density [5,6,7,8], they can be categorized into high-density (ρ > 6.5 g/cm^3^), lightweight (4 g/cm^3^ < ρ < 6.5 g/cm^3^), and ultralight alloys (ρ < 4 g/cm^3^). Alternatively, based on crystal structure [9,10], they can be divided into single-phase solid solutions and multiphase MPEAs. Each classification method is based on the alloy’s structural characteristics or intended application. For example, high-density MPEAs composed of elements like Hf, Mo, Nb, Ni, and Ta typically exhibit high density, high melting points, and high strength, making them suitable for high-temperature applications [11,12,13,14]. Conversely, MPEAs composed of lower-density elements such as Al, Mg, Ti, and V exhibit low density and high specific strength, making them ideal for aerospace and other applications where lightweight and high strength are desired [15,16,17,18].

For lightweight MPEAs with densities ranging from 4 to 6.5 g/cm^3^ [18,19,20], current research primarily uses titanium alloys as reference materials [21,22,23,24,25], aiming to develop novel alloys that surpass traditional titanium alloys in certain performance aspects through compositional design and microstructural control. In summary, research mainly focuses on two categories: TiVCr-based [26] and TiNbZr-based [27] systems. In these systems, Ti and Zr elements are primarily used to form a single-phase BCC structure [28], with Ti also contributing to a reduced alloy density [29]. The addition of V and Nb primarily promotes the formation of the BCC phase and ordered phases [30], with V also enhancing the alloy’s strength [31]. Cr mainly facilitates the formation of the B2 phase and improves the alloy’s workability [32]. Additionally, elements such as Al, Mo, and Mn are incorporated to further adjust the phase composition and enhance performance [24,33,34,35]. For lightweight MPEAs, one of the main challenges is the lack of systems with significantly superior advantages that can completely surpass traditional titanium alloys, indicating that these alloys are still in the compositional design and exploration stage [36]. Moreover, the diverse lightweight MPEA systems, along with their various microstructures and strengthening mechanisms, lack in-depth research, which restricts the further development of this alloy category [37].

To address this issue, this study designs a lightweight MPEA with high strength, ultra-high specific strength, and good ductility, with a molar composition of Ti_61_Al_16_Cr_10_Nb_8_V_5_ (hereafter referred to as Ti61V5 alloy). By conducting an in-depth investigation of the microstructural evolution of Ti61V5 alloy during quasi-static and dynamic plastic deformation processes, this study analyzes the variation in its strengthening mechanisms with strain rate, elucidates the origins of its ultra-high strength and good ductility, and provides a research foundation to support the application and further development of this alloy system.

## 2. Experimental Materials and Methods

### 2.1. Experimental Materials

In this study, a lightweight MPEA with a molar composition of Ti_61_Al_16_Cr_10_Nb_8_V_5_ was synthesized using sponge titanium, industrial pure aluminum, and elemental chromium, niobium, and vanadium particles as raw materials. The alloy was prepared via vacuum levitation melting in a vacuum levitation melting furnace. The elements were added sequentially in order of increasing melting points: Al, Ti, Cr, V, and Nb. Melting was performed six times under an argon atmosphere at a vacuum level of 5 × 10^−4^ Pa, with the ingot flipped and remelted after each cycle to ensure compositional homogeneity. The as-cast Ti61V5 alloy ingot underwent solution treatment at 1000 °C for 8 h. Subsequently, the ingot was sectioned into rectangular billets measuring 4 × 4 × 5.5 cm^3^. These billets were subjected to hot rolling at 980 °C, with a total strain of 90% at a strain rate of 0.005/s. Post-rolling, the samples were aged at 450 °C for 1 h, resulting in Ti61V5 alloy plates approximately 5.5 mm thick.

### 2.2. Experimental Methods

For microstructural analysis, multiple specimens measuring 8 × 8 × 1 cm^3^ were extracted perpendicular to the normal direction (ND) of the hot-rolled Ti61V5 alloy. The samples were ground using abrasive papers, polished with flannel cloth, and etched using a solution comprising 70% water, 20% nitric acid, and 10% hydrofluoric acid by volume. The etched specimens were examined using optical microscopy (OM), scanning electron microscopy (SEM), transmission electron microscopy (TEM), X-ray diffraction (XRD), and electron backscatter diffraction (EBSD). OM analyses were conducted using a Zeiss Axioscope 5 microscope (Jena, Germany), while SEM observations were performed with a Zeiss GeminiSEM 460 (Jena, Germany). For EBSD analysis, samples were electropolished in a solution of 10% perchloric acid, 20% butyl cellosolve, and 70% methanol at 20 V. EBSD measurements were carried out using a Zeiss Sigma 560 microscope (Jena, Germany) with a step size of 3 μm and a resolution of 400 × 400 pixels. TEM samples were prepared by electropolishing in a solution of 10% perchloric acid and 90% methanol at 15 V, followed by observation using an FEI Talos L120C transmission electron microscope (Hillsboro, OR, USA).

Mechanical testing specimens were extracted from the Ti61V5 alloy perpendicular to the transverse direction (TD), aligned along the tensile and compressive directions. The tensile specimens were miniature beams measuring 10 × 2 × 1 mm^3^, while the compression specimens were cylinders with dimensions of Φ3 × 3 mm^3^. Tensile and compression tests were conducted using an INSTRON 6800 universal testing machine (Norwood, MA, USA) at strain rates of 0.001/s, with compression strain rates ranging from 0.001/s to 10/s and total strains of 5%, 30%, and 50%. Dynamic compression tests were performed using an ATL 1200 split Hopkinson pressure bar at strain rates of 1000/s, 3000/s, and 5000/s. Post-mechanical testing, the specimens were sectioned and subjected to OM, SEM, EBSD, and TEM analyses to investigate the microstructural evolution.

**Figure 1:**
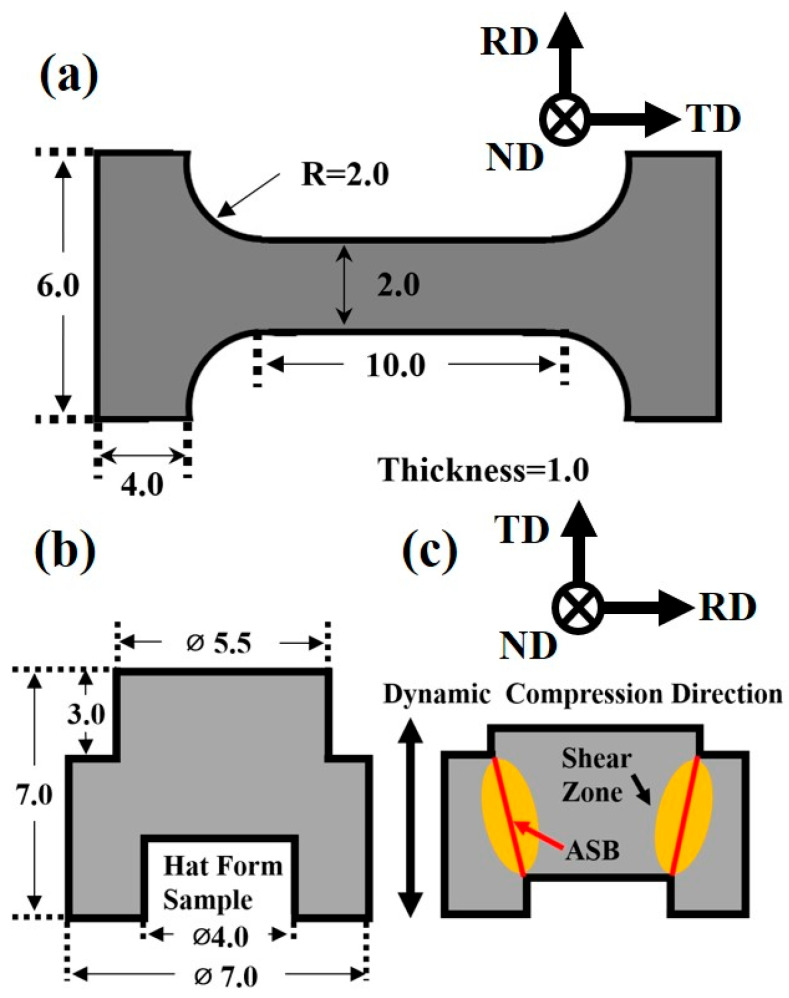
(**a**) Schematic of the tensile specimen dimensions; (**b**) Dimensions of the forced shear specimen; (**c**) Schematic illustration of the forced shear specimen.

Mechanical testing specimens were extracted from the Ti61V5 alloy, and both the tensile and compressive loading axes were aligned parallel to the transverse direction (TD). The tensile specimens were miniature beams with dimensions of 10 × 2 × 1 mm^3^, while the compression specimens were cylindrical samples with dimensions of Φ3 × 3 mm^3^. The forced shear specimens were prepared according to the geometry shown in Figure 1. Tensile and compression tests were conducted using an INSTRON 6800 universal testing machine at strain rates of 0.001/s, with compression strain rates ranging from 0.001/s to 10/s and total strains of 5%, 30%, and 50%. Dynamic compression and forced shear tests were carried out using an ATL 1200 split Hopkinson pressure bar system, with dynamic compression strain rates of 1000/s, 3000/s, and 5000/s, and forced shear tests conducted at fixed strains of 5–20%. After mechanical testing, the specimens were sectioned and subjected to OM, SEM, EBSD, and TEM analyses to examine the microstructural characteristics.

## 3. Experiment Results

### 3.1. Microstructure and Mechanical Properties of Ti61V5 Alloy

**Figure 2:**
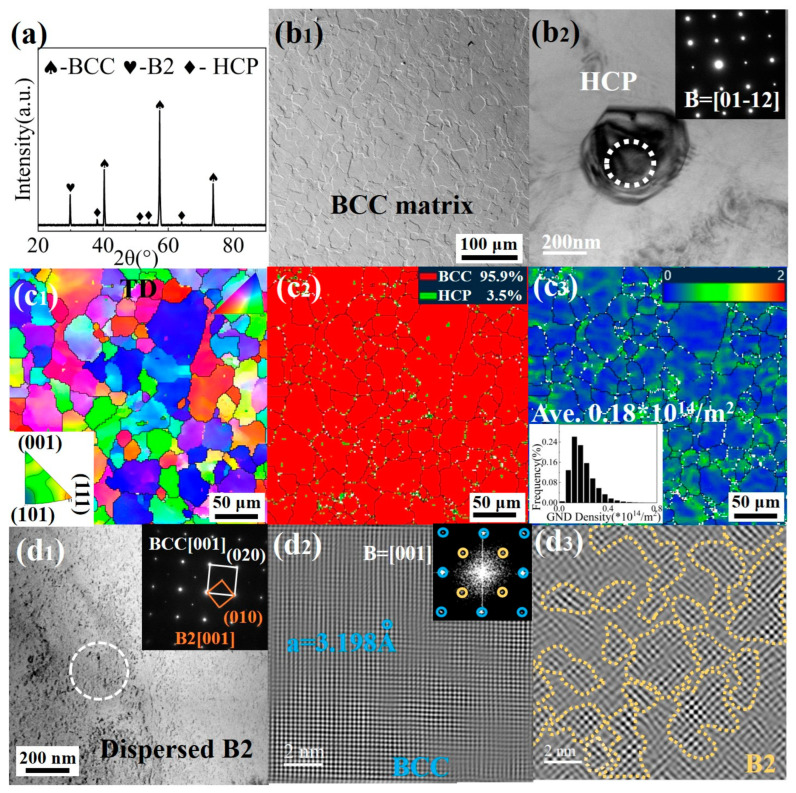
Microstructure of the Ti_61_Al_16_Cr_10_Nb_8_V_5_ alloy in the initial state: (**a**) XRD pattern; (**b_1_**) SEM image of BCC; (**b_2_**) TEM image of HCP; (**c_1_**) EBSD inverse pole figure; (**c_2_**) phase distribution map; (**c_3_**) GND map; (**d_1_**) TEM image and SAED of BCC/B2 phases; (**d_2_**,**d_3_**) SAED and IFFT of BCC/B2 phases.

The microstructure of the Ti61V5 alloy after hot-worked processing was examined, and the results are presented in Figure 2. As shown in Figure 2a, the alloy predominantly consists of a BCC phase, with minor amounts of B2 phase and trace diffraction peaks corresponding to HCP phases. The morphological observations in Figure 2(b_1_,b_2_) reveal that the BCC phase exhibits an equiaxed morphology, with grain interiors containing a few spherical HCP phases. The EBSD results in Figure 2(c_1_–c_3_) indicate that the average grain size of the BCC matrix is approximately 35 μm, with no evident texture. Phase distribution maps show that the combined volume fraction of HCP phases is about 3.5%, primarily located at BCC grain boundaries, with a small amount dispersed within the grains. Geometrically necessary dislocation (GND) analysis reveals an average dislocation density of approximately 0.18 × 10^14^ m^−2^, indicating a relatively low overall density, with higher densities concentrated at BCC grain boundaries and BCC/HCP phase interfaces. The TEM observations in Figure 2(d_1_–d_3_) demonstrate the presence of B2 superlattice reflections throughout the BCC matrix, suggesting that the B2 phase is finely dispersed within the BCC matrix and maintains a coherent relationship. The inverse fast Fourier transform (IFFT) of selected area electron diffraction patterns (SAED) for the BCC/B2 phases in Figure 2(d_1_–d_3_) indicates that the B2 phase is dispersed within the BCC matrix in an irregular form, ranging from a few nanometers to several tens of nanometers. The lattice constant of the BCC phase is determined to be a_(BCC)_ = 3.198 Å. Furthermore, the density of the Ti61V5 alloy, measured using the Archimedes drainage method, is approximately 4.82 g/cm^3^.

**Figure 3:**
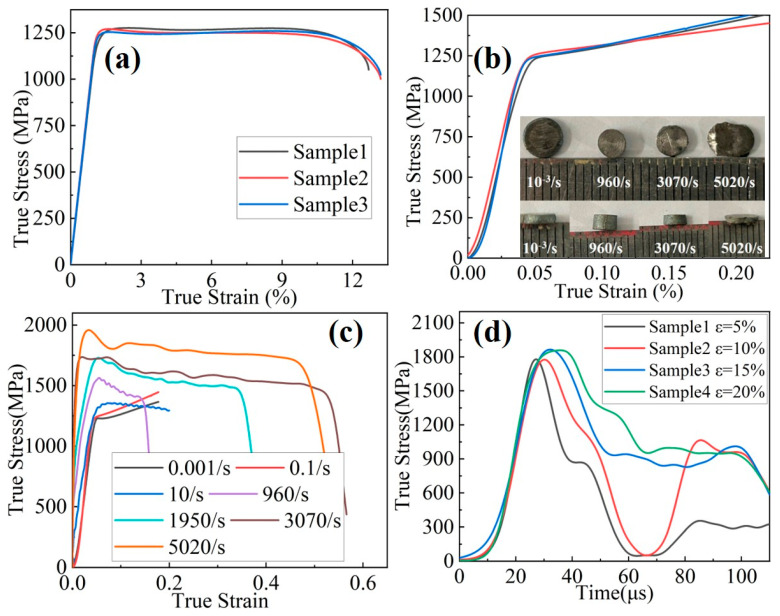
Mechanical properties of the Ti_61_Al_16_Cr_10_Nb_8_V_5_ alloy: (**a**) quasi-static tensile properties; (**b**) quasi-static compressive properties and post-compression morphologies under different conditions; (**c**) compressive properties at various strain rates; (**d**) shear stress–time curves under forced shear test.

Quasi-static mechanical tests were conducted on the Ti61V5 alloy, and the results are presented in Figure 3a,b. Under quasi-static conditions, the alloy exhibited a yield strength of approximately 1110 ± 25 MPa, an ultimate tensile strength of about 1260 ± 18 MPa, and a tensile elongation of approximately 12.3 ± 0.5%. In compression, the yield strength was around 1200 ± 33 MPa, with a compressive strain exceeding 50%. Considering the alloy’s density, the specific tensile strength was calculated to be approximately 262 MPa·cm^3^/g. These quasi-static mechanical properties indicate that the Ti61V5 alloy possesses high strength, ultra-high specific strength, and good ductility.

Compression tests were conducted on the Ti61V5 alloy at various strain rates, and the results are shown in Figure 3c. It can be observed that the flow stress of the alloy increases with an increasing strain rate. At a strain rate of 3070/s, the alloy exhibits a flow stress of 1630 MPa and sustains a strain of up to 50% without fracture. When the strain rate is increased to 5020/s, the flow stress rises to 1810 MPa, and the alloy undergoes 45° shear fracture at approximately 50% strain. These dynamic compression results indicate that the alloy exhibits a pronounced strain-rate-strengthening effect and excellent dynamic compressive ductility. The post-deformation morphology of the alloy after dynamic compression is shown in Figure 3b.

To further investigate the strain-rate-strengthening effect of the Ti61V5 alloy, compressive stress values at various strain rates were statistically analyzed, with the results presented in Table 1. Additionally, the strain rate sensitivity coefficient m was introduced, defined in Equation (1) as follows [38]:

(1) $$m=\frac{\partial ln\;\sigma }{\partial ln\;\dot{\varepsilon }}$$

The symbol σ denotes stress, and $\dot{\varepsilon }$ represents the strain rate. Based on Equation (1), the strain rate sensitivity coefficient *m* was calculated for the yield strength, ultimate tensile strength, and flow stress of the Ti61V5 alloy. It was found that at low strain rates ranging from 0.001/s to 10/s, the average *m* value was approximately 0.0088. However, when the strain rate increased to the range of 960/s to 5020/s, *m* increased significantly to an average value of 0.0985. This notable increase in strain rate sensitivity indicates a change in the dominant deformation mechanism of the alloy.

Forced shear tests were also conducted on the Ti61V5 alloy at fixed strains of 5%, 10%, 15%, and 20%, and the resulting shear stress–time curves are shown in Figure 3d. As seen from the figure, both the peak stress and the corresponding time increase slightly with increasing strain. This rise in peak stress is attributed to the strain-rate-strengthening effect. In addition, the second stress plateau observed in the curves is caused by the limiting ring.

### 3.2. Plastic Deformation Mechanisms of Ti61V5 Alloy at Low Strain Rates

**Figure 4:**
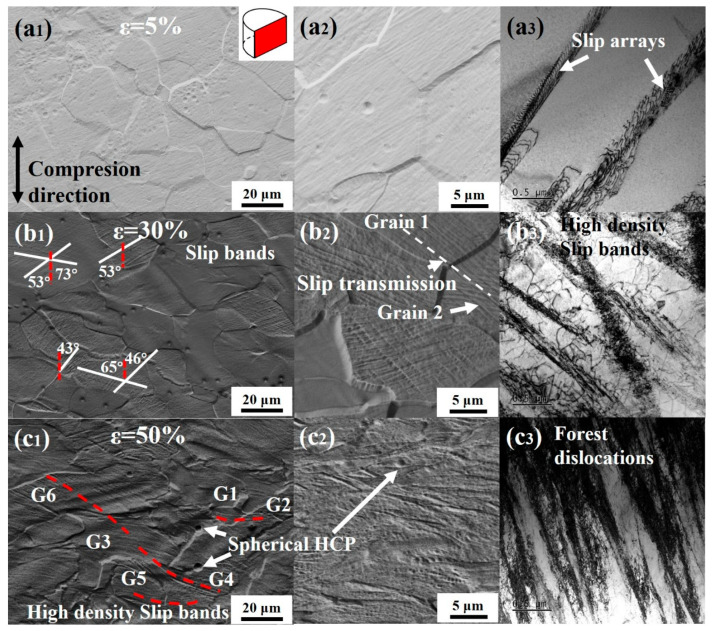
Microstructures of Ti_61_Al_16_Cr_10_Nb_8_V_5_ alloy after quasi-static compression at fixed strains: (**a**) ε = 5%; (**b**) ε = 30%; (**c**) ε = 50%; (**1**) Low-magnification SEM images; (**2**) High-magnification SEM images; (**3**) TEM images.

To elucidate the plastic deformation mechanisms and their evolution in the Ti61V5 alloy under varying conditions, microstructural observations were conducted on specimens subjected to quasi-static compression at fixed strains of ε = 5%, 30%, and 50%. The results are presented in Figure 4, with the observation locations indicated by the red cross-sections in the schematic diagram. As shown in Figure 4(a_1_–a_3_), at a low strain level (ε = 5%), the BCC grains of the alloy exhibit no significant deformation features. A few dislocation arrays are observed within the grains, which are attributed to the dislocations cutting through the ordered B2 phase [39]. When the strain increases to 30%, as depicted in Figure 4(b_1_), numerous slip bands become visible under SEM, traversing entire BCC grains. Some grains contain two or even three sets of slip bands in different orientations, which intersect with each other. Most slip bands terminate at grain boundaries; however, a few propagate across the boundaries into adjacent grains, as shown in Figure 4(b_2_). Measurements indicate that the majority of slip bands are oriented at approximately 45° to the compression direction, corresponding to the direction of maximum Schmid factor [40]. TEM observations reveal a significant increase in dislocation density within the alloy. An abundance of slip bands is observed, with frequent intersections among them. Additionally, numerous curved dislocations not confined within slip bands are present, which are believed to result from the interactions of high-density slip bands [41] and Suzuki segregation effects [42]. At a strain of 50%, the BCC grains undergo severe compressive deformation, and the density of slip bands within the grains further increases. The number of slip bands transmitting across grains also rises markedly. TEM analysis shows that numerous slip bands intersect and entangle, forming high-density dislocation forests.

**Figure 5:**
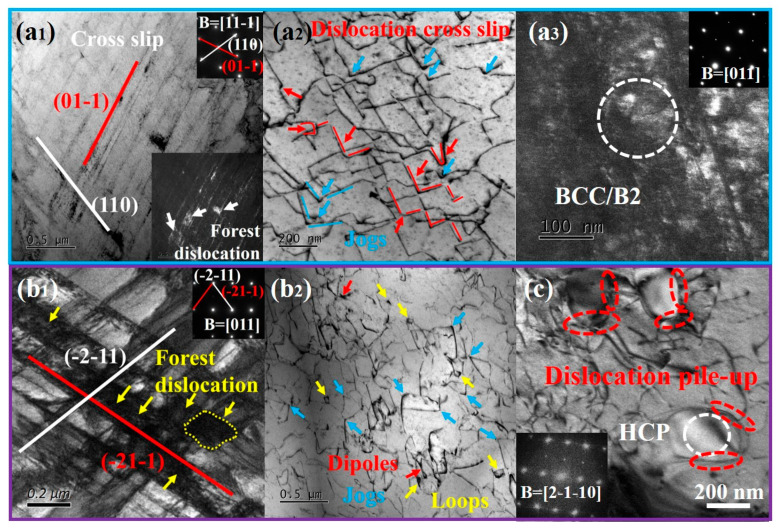
Microstructures of Ti_61_Al_16_Cr_10_Nb_8_V_5_ alloy after Fixed-strain compression: (**a_1_**–**a_3_**) Dislocation structures within BCC/B2 grains at ε = 30%; (**b_1_**,**b_2_**) Dislocation structures within BCC/B2 grains at ε = 50%; (**c**) Morphology of HCP phase at ε = 50%.

To further investigate the plastic deformation mechanisms of the Ti61V5 alloy under quasi-static compression, TEM observations were conducted on specimens compressed to strains of 30% and 50%. The results are presented in Figure 5. At a strain of 30%, as shown in Figure 5(a_1_–a_3_), the BCC grains exhibit numerous straight lines lying on the (01-1) and (110) planes, which are identified as projected traces of the {011} slip planes, indicating that planar slip predominantly occurs on the {011} densely packed planes [43]. At the intersections of different slip bands, prominent dislocation forests are observed, suggesting interactions between intersecting slip bands. Figure 5(a_2_) reveals the presence of numerous sharp features within the BCC grains, where straight dislocations are bent into L-shaped and U-shaped configurations. These sharp features are characteristic of dislocation jogs [44], formed when screw dislocations on one plane are intersected by edge dislocations or screw dislocations on another plane, indicative of cross-slip phenomena [44,45]. The presence of multiple L-shaped and U-shaped structures serves as strong evidence for double cross-slip events [46]. Additionally, Figure 5(a_3_) demonstrates that dislocations have cut through the B2 phase. At a higher strain of 50%, as depicted in Figure 4(c_1_–c_3_)the alloy exhibits further evolution in its deformation mechanisms. Figure 5(b_1_) shows that, in addition to the {011} planes, slip bands begin to appear on the {112} planes, both of which are primary slip planes in BCC crystals [47]. The number of dislocation forests at the intersections of slip bands increases significantly. Figure 5(b_2_) indicates that, besides the formation of numerous dislocation jogs, a small number of dislocation loops and dipoles are also present. Dislocation dipoles are generally formed by the interaction of two oppositely signed edge dislocations during cross-slip, and their subsequent encounter can lead to the formation of dislocation loops [44]. Therefore, the presence of dislocation dipoles and loops provides strong evidence for extensive cross-slip activity. The deformation behavior of the HCP phase was examined, as shown in Figure 5c. It is evident that the dislocation density within the HCP phase is significantly lower than that in the BCC matrix, and noticeable dislocation pile-ups occur at the phase boundaries. This indicates that the HCP phase strengthens the alloy through a dislocation bypass mechanism.

**Figure 6:**
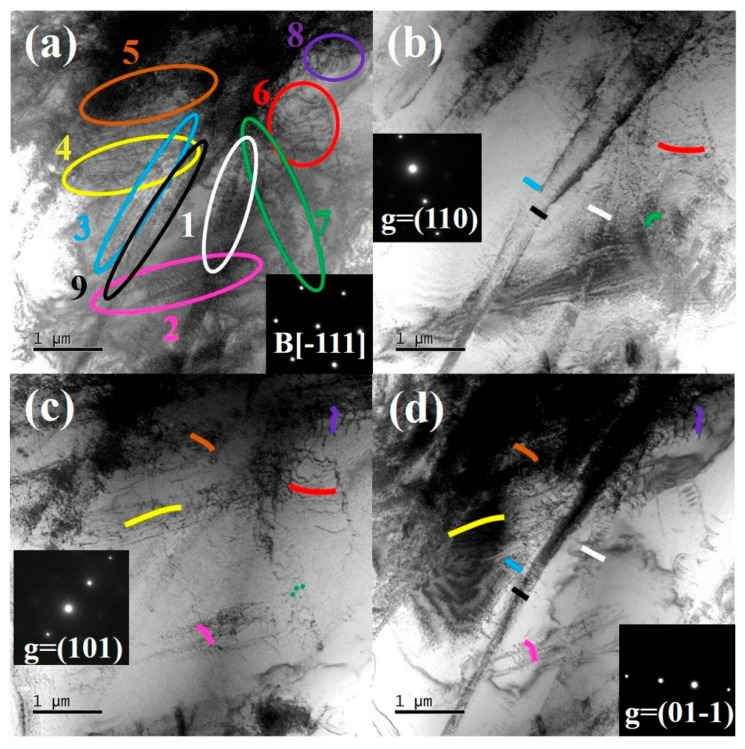
Dislocation contrast images of Ti_61_Al_16_Cr_10_Nb_8_V_5_ alloy after 50% strain quasi-static compression under different g vectors: (**a**) Overall morphology with B = [-111]; (**b**) g = (110); (**c**) g = (101); (**d**) g = (01-1).

To elucidate the dislocation types present in the Ti61V5 alloy subjected to 50% strain under quasi-static compression, TEM analyses were conducted using different incident electron beam directions and g vectors. The results are depicted in Figure 6 (B = [-111]) and Supplementary Figure S1 (B = [011]). As shown in Figure 6, dislocations labeled 1, 2, 3, 6, and 9 form continuous dislocation arrays, while the remaining dislocations are isolated. The extinction conditions of these dislocations under various g vectors are summarized in Table 2. By analyzing the extinction behavior and the projected images under different incident beam directions, the Burgers vectors, dislocation types, and line directions for dislocations 1 through 9 were determined, as presented in Table 3. An analysis of Table 3 reveals that the majority of dislocations in the Ti61V5 alloy are of mixed character. Notably, according to the statistical results, all dislocations except dislocations 1 and 4 are mixed dislocations dominated by screw character. This prevalence of screw dislocations is attributed to their lower formation energy and critical resolved shear stress, as well as their ability to glide on multiple slip systems, making them more likely to form during quasi-static deformation [48]. Furthermore, observations from Figure 5c indicate that the HCP phase strengthens the alloy through a dislocation bypass mechanism. Consequently, when dislocations within BCC grains encounter obstacles such as dislocation forests or precipitates during deformation, some dislocations accumulate at these interfaces, while others, upon receiving additional energy, bypass the obstacles via screw dislocation cross-slip or edge dislocation climb. These mechanisms contribute to the formation of numerous mixed dislocations and facilitate the bypassing of precipitates, thereby enhancing the alloy’s mechanical properties.

### 3.3. Plastic Deformation Mechanisms of Ti61V5 Alloy at High Strain Rates

**Figure 7:**
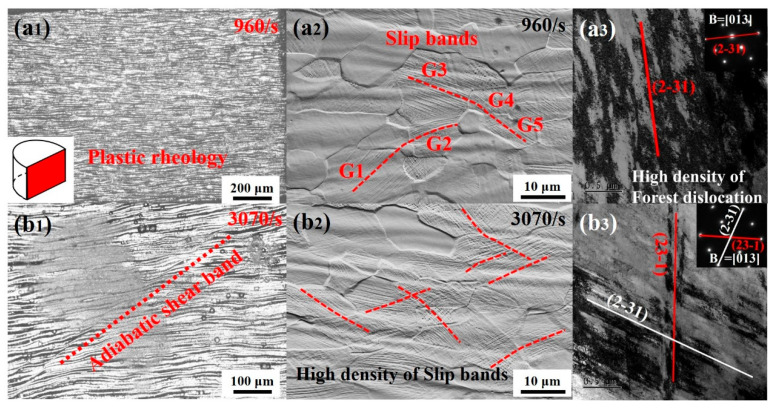
Microstructures of the Ti_61_Al_16_Cr_10_Nb_8_V_5_ alloy under high strain rates: (**a**) Morphology at a strain rate of 960/s; (**b**) Morphology at a strain rate of 3070/s; (**1**) OM morphology; (**2**) SEM morphology; (**3**) TEM morphology and dark-field image.

The microstructures of the Ti61V5 alloy subjected to compression tests at strain rates of 960/s and 3070/s were investigated, and the obtained results are presented in Figure 7. As shown in Figure 7a, at a strain rate of 960/s, significant plastic deformation occurred in the alloy, exhibiting a pronounced wavy morphology indicative of plastic flow. A high density of slip bands appeared within grains, intersecting with each other, and a number of slip bands underwent transgranular transmission. TEM observations revealed that dislocations formed slip bands not only on {011} and {112} crystal planes but also on higher-index planes {123}. Interactions among slip bands from different crystallographic planes at their intersections resulted in the formation of numerous dislocation forests. When the strain rate further increased to 3070/s (Figure 7b), the alloy similarly exhibited plastic flow characteristics accompanied by the appearance of a small number of shear bands approximately 5–10 μm wide. These shear bands originated from the combined effects of local stress concentration and thermal softening during high-speed deformation [49]. However, their sparse distribution and relatively narrow dimensions indicated a low susceptibility to adiabatic shear in the Ti61V5 alloy, beneficial for its plasticity. Additionally, an increased density of slip bands within BCC grains was observed, with many slip bands undergoing transgranular slip. Further observations reveal that a large number of slip bands also form on higher-index planes such as {112} and {123}, and the extensive interactions among these slip bands further increase the density of dislocation forests.

**Figure 8:**
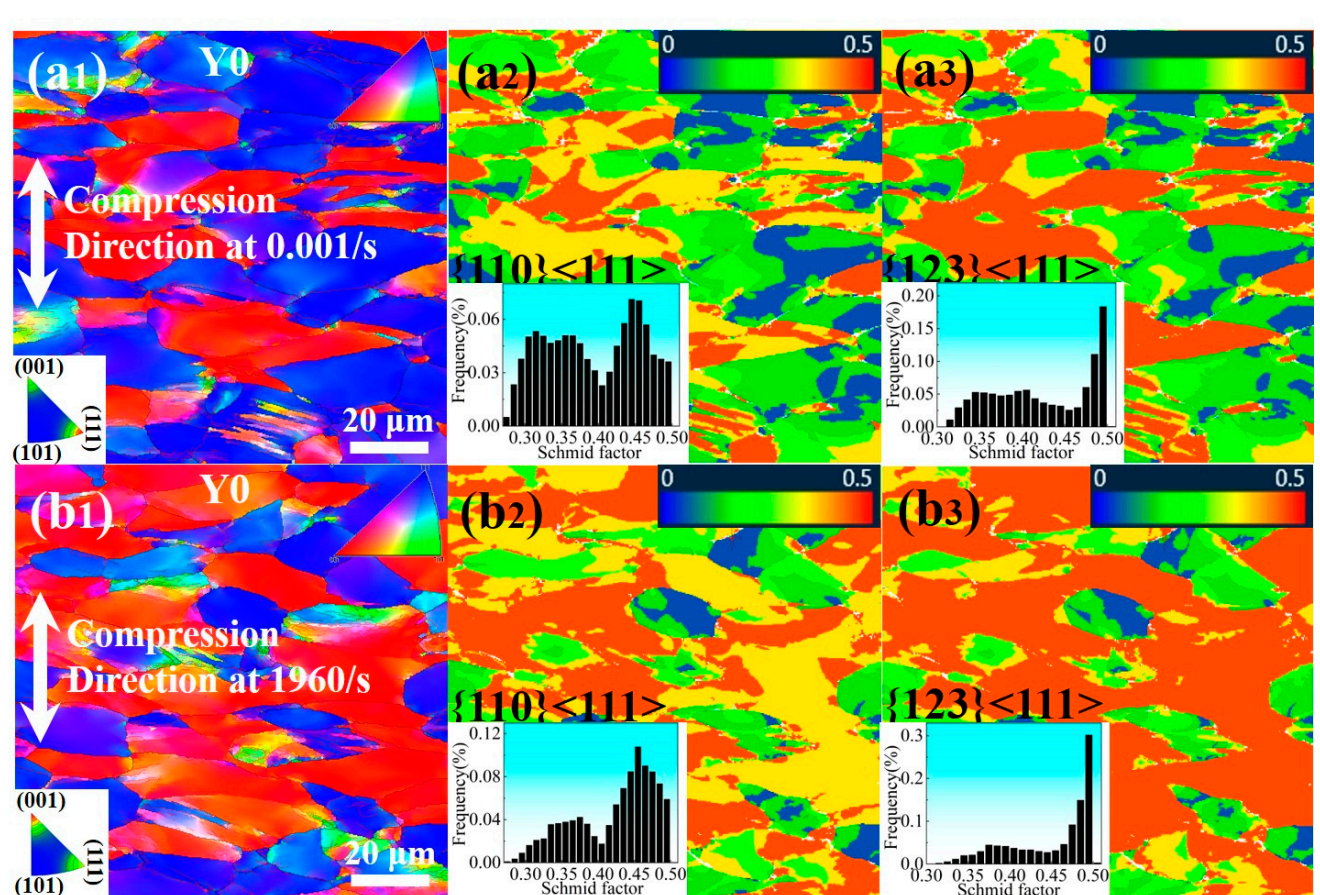
EBSD results of the Ti_61_Al_16_Cr_10_Nb_8_V_5_ alloy after 30% strain under different strain rates: (**a**) EBSD results under quasi-static compression; (**b**) EBSD results under a strain rate of 1950/s; (**1**) Inverse pole figures; (**2**) Schmid factor distribution maps for the {110}<111> slip system; (**3**) Schmid factor distribution maps for the {123}<111> slip system.

To further verify the evolution of slip systems in the BCC matrix of the Ti61 alloy under quasi-static and high-strain-rate conditions, microstructural observations were conducted on specimens compressed to a fixed strain of 30% under quasi-static loading and to an engineering strain of 30.7% at a strain rate of 1950/s. The results are shown in Figure 8. As seen in Figure 8(a_1_–a_3_), significant grain orientation was observed in the alloy after compression under both quasi-static and high-strain-rate conditions. A large number of {001} and {111} planes rotated to become nearly perpendicular to the compression direction (Y0 axis). Furthermore, under the high strain rate of 1950/s, as shown in Figure 8(b_3_), the fraction of grains with high Schmid factors (0.45–0.5) for the high-index {123}<111> slip system reached approximately 79%, which is notably higher than the 61% observed under quasi-static conditions, as shown in Figure 8(a_3_). This indicates that as the strain rate increases, the alloy’s ability to activate the high-index {123}<111> slip bands is significantly enhanced, which is consistent with the TEM observations.

**Figure 9:**
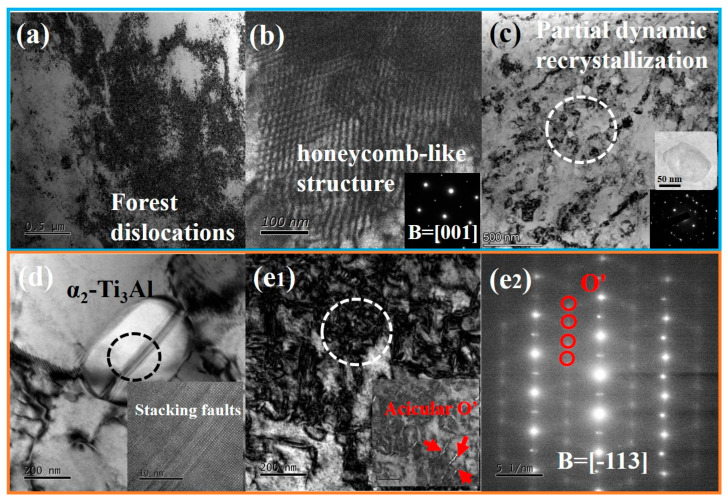
TEM morphologies of the Ti_61_Al_16_Cr_10_Nb_8_V_5_ alloy after dynamic compression: (**a**) Dislocation structures at 3070/s; (**b**) Dislocation structures at 5070/s; (**c**) Incomplete dynamic recrystallization and HRTEM at 5070/s; (**d**) α_2_-Ti_3_Al phase and stacking faults at 5070/s; (**e_1_**,**e_2_**) O′ phase transformation: bright/dark-field image and SAED at 5070/s.

Further TEM characterization of the dynamically compressed Ti61V5 alloy was conducted, and the results are presented in Figure 9. As shown in Figure 9a, a high density of dislocation forests formed in the alloy at a strain rate of 3070/s. This phenomenon is attributed to extensive cross-slip among multiple slip bands and the entanglement of dislocations during the climb of edge dislocations. When the strain rate increased to 5020/s, a honeycomb-like dislocation structure appeared within certain BCC grains, as shown in Figure 9b. This structure is considered to be associated with dislocation climb and other recovery processes [50]. Figure 9c shows that some BCC grains exhibited equiaxed new BCC subgrains with diameters of approximately 100–200 nm. These are believed to be formed via incomplete dynamic recrystallization under the instantaneous high-temperature and high-pressure environment at 5020/s, suggesting the occurrence of rapid dynamic recrystallization in localized regions. Moreover, a small number of dislocations and stacking faults were observed within the HCP phase (Figure 9d), indicating that the HCP phase also experienced significant plastic deformation under such conditions. In addition, a small number of needle-like structures were observed within certain BCC grains (Figure 9(e_1_,e_2_)), with widths of ~10 nm and lengths of 100–300 nm. SAED analysis identified superlattice reflections characteristic of the O′ phase on the 1/2(1-21) plane along the [-113] zone axis of the BCC matrix [51,52]. This confirms that the needle-like structures are O′ phase precipitates. The O′ phase is a non-equilibrium orthorhombic phase generally regarded as a martensitic transformation product, formed by atomic shuffling along the {011}[011] slip system of BCC under stress [53,54]. Owing to its high resistance to plastic deformation, the presence of the O′ phase can significantly enhance the strength of the Ti61V5 alloy.

**Figure 10:**
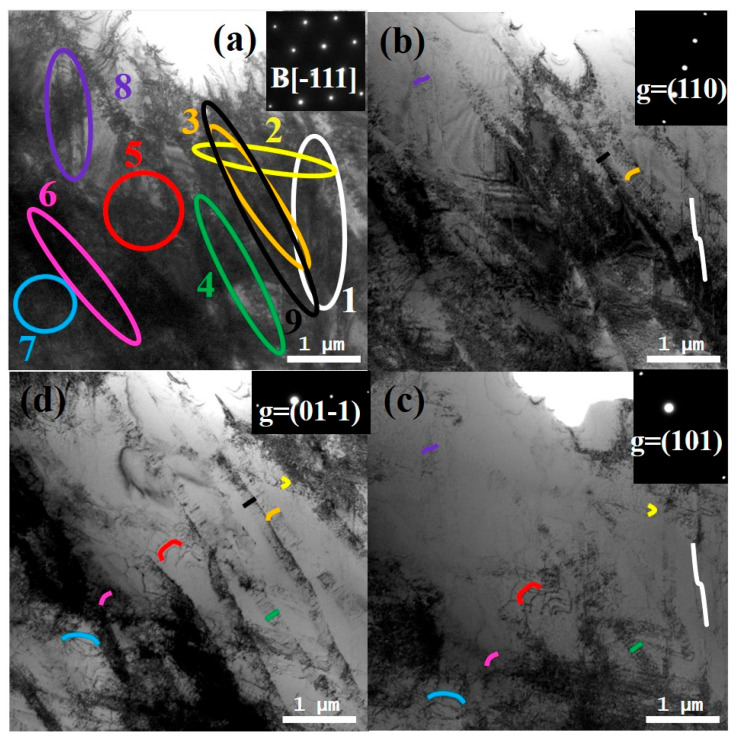
Dislocation extinction images of the Ti_61_Al_16_Cr_10_Nb_8_V_5_ alloy after compression at a strain rate of 5020/s under different g vectors: (**a**) Overall morphology with B = [-111]; (**b**) g = (110); (**c**) g = (101); (**d**) g = (01-1).

Dislocation character analysis was performed on the Ti61V5 alloy compressed at a strain rate of 5020/s. The results are shown in Figure 10 (B = [-111]) and Supplementary Figure S2 (B = [011]). Based on Figure 10, dislocations 2, 3, 4, 6, and 9 were identified as dislocation arrays, while dislocations 1, 5, 7, and 8 were determined to be isolated dislocations. By combining the extinction criterion (Table 2) with the analysis in Supplementary Figure S2, the Burgers vectors, line directions, and types of these dislocations were identified. The results are summarized in Table 4. As shown in the table, mixed dislocations still dominated in the Ti61V5 alloy after high-strain-rate compression at 5020/s. However, according to the statistical results, a noticeable increase in the proportion of edge-character-dominated mixed dislocations was observed compared with quasi-static deformation, with all dislocations except dislocation 9 being dominated by edge character. This is believed to result from the high-temperature and high-pressure environment generated during dynamic compression. The instantaneous energy input is sufficient to induce the formation of a large number of edge dislocations and promote their climb, thereby increasing their proportion. This rise in the edge dislocation content contributes positively to the plasticity of the alloy during dynamic deformation.

### 3.4. Shear Band and Crack Evolution in Ti61V5 Alloy After Forced Shear Deformation

**Figure 11:**
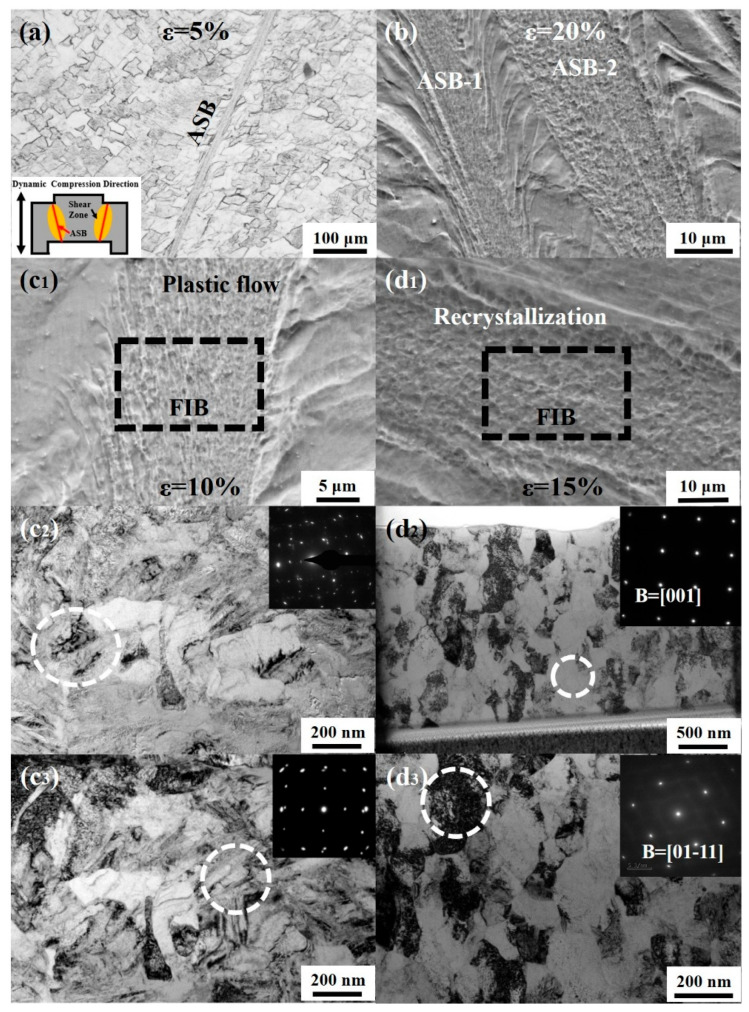
Shear band morphologies of the Ti_61_Al_16_Cr_10_Nb_8_V_5_ alloy after forced shear deformation: (**a**) Shear band morphology at ε = 5%; (**b**) Shear band morphology at ε = 20%; (**c_1_**–**c_3_**) SEM and TEM images of a plastic-flow shear band at ε = 10%; (**d_1_**–**d_3_**) SEM and TEM images of a recrystallized shear band at ε = 15%.

The dynamic compression results indicate that shear bands in the Ti61V5 alloy only begin to form at strain rates of 3070/s and above, suggesting the alloy possesses low sensitivity to adiabatic shear localization. This characteristic benefits its compressive performance but also makes it difficult to observe the shear fracture mechanism during dynamic compression. Therefore, forced shear tests at fixed strains of 5% to 20% were conducted on the Ti61V5 alloy to enable a microstructural observation of the resulting shear bands, as shown in Figure 11. Figure 11a,b show that at 5% strain, only a single shear band approximately 10–20 μm in width is observed in the shear region. As the strain increases to 20%, multiple shear bands with similar widths appear, indicating that the number of shear bands increases with strain. Additionally, two morphologically distinct types of shear bands are observed. Under low strain, the dominant type is shown in Figure 11(c_1_), characterized by deformation resembling a fluid-like state within the band. FIB sampling and subsequent analysis, as shown in Figure 11(c_1_,c_2_), reveal that the grains inside the shear band undergo severe plastic deformation, with many grains transforming into amorphous structures and a small number of deformation twins present. These are referred to as plastic-flow shear bands. At higher strains, besides plastic-flow shear bands, a second type of shear band appears, as illustrated in Figure 11(d_1_). These bands contain nanometer-sized spherical grains. After FIB sampling, TEM observation was carried out, and the results in Figure 11(d_2_,d_3_) show equiaxed grains ranging from 100 to 500 nm, some of which are free from apparent dislocation structures or defects. SAED confirms that most of these grains possess a BCC structure, with a few being HCP. This type is referred to as a recrystallized shear band.

**Figure 12:**
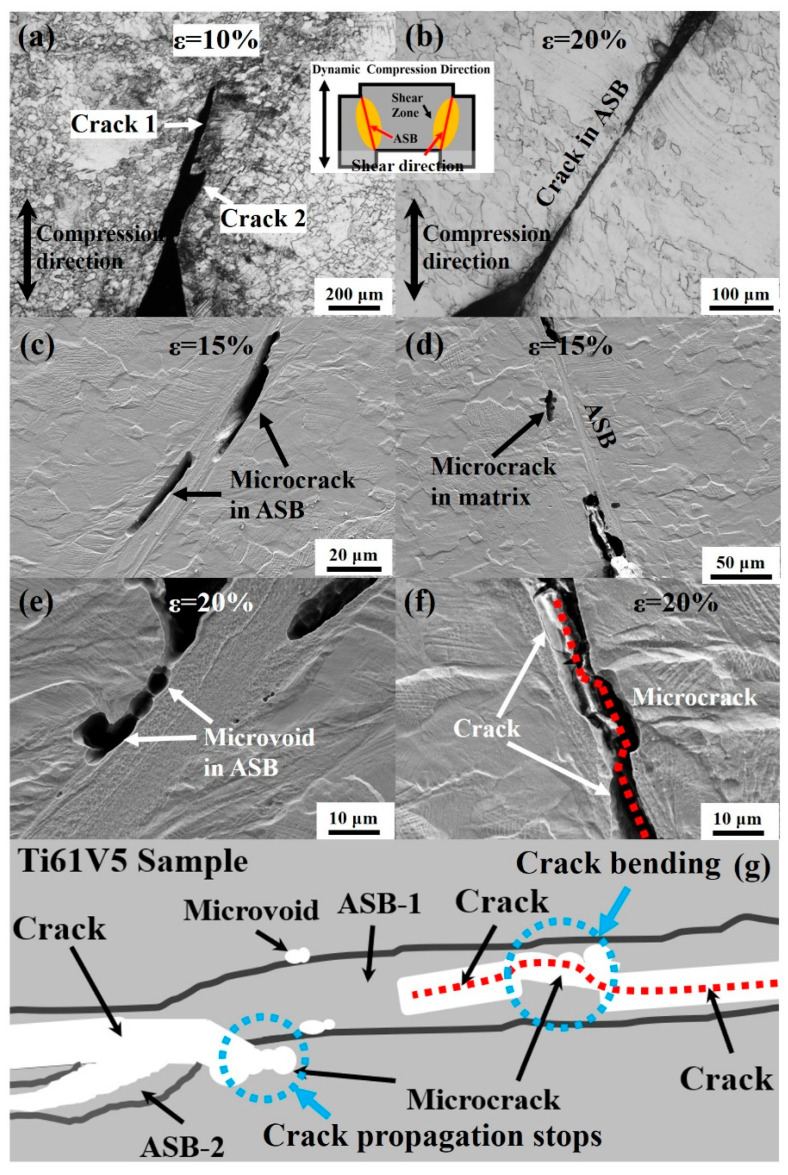
Shear crack morphologies of the Ti_61_Al_16_Cr_10_Nb_8_V_5_ alloy after forced shear deformation: (**a**) Shear cracks at ε = 10%; (**b**) Shear cracks at ε = 20%; (**c**,**d**) Microcracks at ε = 15%; (**e**,**f**) Microvoids and microcracks at ε = 20%; (**g**) Schematic illustration of crack propagation within the shear band.

The shear cracks of the Ti61V5 alloy after forced shear deformation were examined, and the results are shown in Figure 12. As shown in Figure 12a, cracks begin to form at the initial end of the shear zone at a strain of 10%, initiating and propagating within the shear bands. When the strain increases to 20%, some cracks propagate along the shear bands, eventually traversing the entire shear region, as shown in Figure 12b. Observations of the shear zone at 15% strain, as shown in Figure 12c,d, reveal that in addition to crack initiation at the initial end of the shear region and subsequent inward propagation, microcracks and microvoids also form in the central area of the shear bands. Moreover, a small number of microcracks are found in the matrix near the shear bands. Further observation of the shear zone at 20% strain, as presented in Figure 12e,f, shows that as the primary crack propagates along the shear band, it may terminate upon encountering microcracks or microvoids outside the band. In contrast, when encountering microcracks within the shear band, the crack tends to link up and deflect slightly but continues to propagate. A schematic illustration of the shear crack propagation in the Ti61V5 alloy is shown in Figure 12g.

## 4. Discussion

### 4.1. Evolution of Dislocation Mechanisms in Ti61V5 Alloy Under Varying Strain Rates

Based on microstructural observations of the Ti61V5 alloy under different strain rates, it is evident that the dislocation deformation mechanisms vary significantly between high and low-strain-rate conditions. Under low strain rates ranging from 0.001/s to 10/s, a large number of slip bands along the {011} and {112} planes are observed in the alloy, as shown in Figure 5(a_1_,b_1_). In Ti61V5, the {011} plane is the close-packed plane of the BCC grains and possesses the lowest slip energy barrier, thus activating first during deformation. As strain accumulates, extensive interactions among the slip bands result in the formation of dislocation forests. Meanwhile, other slip bands, together with edge dislocation climb, contribute to the formation of dislocation jogs. The presence of dislocation forests, jogs, and precipitates boundaries significantly hinders dislocation slip on the {011} plane. With continued external energy input during deformation, the {112} slip bands are gradually activated. Due to their similarly low energy barrier, the activation of {112} planes and their interactions with preexisting slip bands—through cross-slip or even double cross-slip—help alleviate dislocation pile-up, thereby avoiding premature stress localization and enabling the alloy to retain high compressive ductility. In addition, dislocation character analysis indicates that most dislocations at low strain rates are mixed in nature, with a dominant screw component. This is attributed to the extensive cross-slip of screw dislocations on multiple slip planes, resulting in the formation of numerous mixed dislocations.

Under high strain rates between 960/s and 5020/s, as illustrated in Figure 7(a_3_,b_3_) and Figure 9a,b, numerous slip bands form within the BCC grains. In addition to those on the {011} and {112} planes, many high-index planes {123} are also activated due to the high-temperature, high-pressure conditions and intense energy input during deformation. Intense interactions among these densely distributed slip bands result in the formation of even higher-density forest dislocations, which significantly hinder further dislocation motion and substantially increase the alloy’s strength under dynamic conditions. This is identified as the primary reason for the pronounced strain-rate-strengthening effect observed in the alloy. Moreover, dislocation type analysis shows that although mixed dislocations remain dominant under dynamic conditions, the proportion of edge-character-dominated mixed dislocations increases substantially. This is primarily attributed to the greater hindrance introduced by the dense dislocation structures, making it difficult for subsequent dislocations on the same plane to bypass obstacles through cross-slip. However, the elevated temperature, pressure, and energy input during dynamic compression facilitate the climb of edge dislocations. As a result, some screw dislocations in the slip bands transform into mixed dislocations via climb and bypass the obstacles, leading to an increased fraction of edge-character dislocations [41]. The formation of numerous high-index slip bands and the increased proportion of edge dislocations enhance the alloy’s ability to bypass obstacles via cross-slip or climb mechanisms. This suppresses early stress concentration and adiabatic shear localization, thereby improving the alloy’s compressive ductility and enabling it to maintain a fracture strain of approximately 50% under dynamic compression.

**Figure 13:**
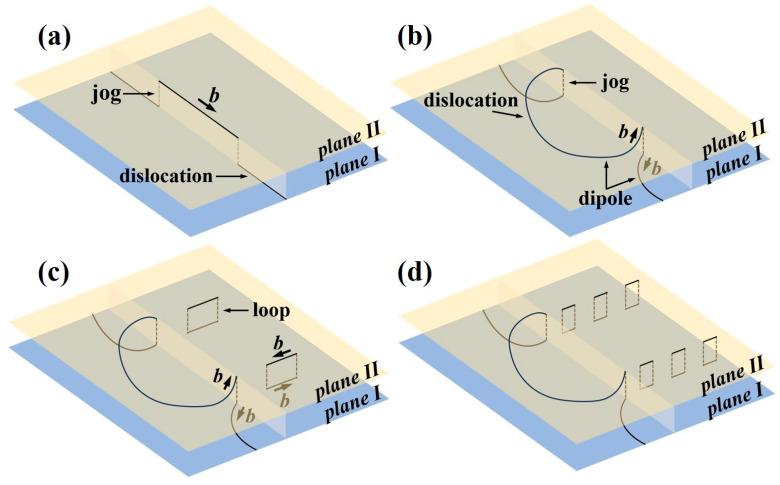
Schematic illustration of dislocation interactions during cross-slip or climb in the alloy: (**a**) Formation of ledges via cross-slip or climb; (**b**) Formation of dislocation dipoles between edge dislocations with opposite Burgers vectors; (**c**) Annihilation of dislocation dipoles leading to the formation of dislocation loops; (**d**) Alignment of dislocation loops into linear arrays [43,44].

In addition, a large number of dislocation arrays were observed in the Ti61V5 alloy after deformation under both high and low strain rates, as shown in Figure 3a, Figure 6 and Figure 10. This phenomenon is attributed primarily to the presence of short-range ordered structures (i.e., B2 phase) in the alloy. As indicated in Figure 2(d_3_) and Figure 5(a_3_), the B2 phase in the Ti61V5 alloy is nanosized and dispersed within the BCC matrix, and dislocations are observed to cut through the B2 phase during slip. Due to the short-range atomic ordering in the B2 phase, which possesses a higher energy barrier compared to the disordered BCC matrix, leading dislocations must overcome a high critical shear stress to initiate slip. However, once a dislocation moves through the B2 phase, it disrupts the ordered atomic arrangement along its slip path, thereby lowering the energy barrier for subsequent dislocations along the same path [39]. As a result, dislocations tend to follow the same slip path and form dense dislocation arrays, as shown in Figure 13 [43,44]. This mechanism is not unique to the Ti61V5 alloy but is commonly observed in alloys containing short-range ordered structures.

### 4.2. Evolution of Deformation Mechanisms in Ti61V5 Alloy Under Varying Strain Rates

**Figure 14:**
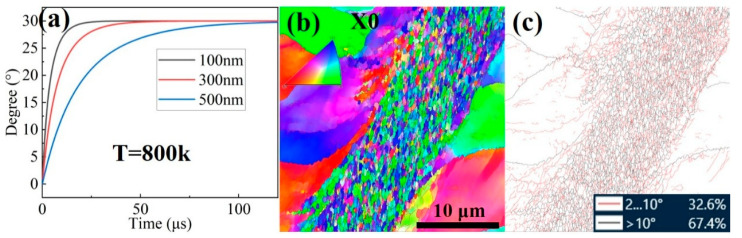
Dynamic recrystallization in the Ti_61_Al_16_Cr_10_Nb_8_V_5_ alloy: (**a**) RDRX angle–time curve; (**b**) morphology of incompletely dynamically recrystallized grains. (**b**) EBSD inverse pole figure; (**c**) Grain boundary distribution map.

In addition to significant changes in the dislocation mechanisms, the Ti61V5 alloy also exhibits distinct differences in other deformation mechanisms under high and low strain rates. At low strain rates, only dislocation forests and dislocation walls are observed within BCC grains, whereas substructures such as dislocation cells are scarcely present. In the HCP phase, a limited number of dislocations gradually appear with increasing strain. In contrast, under high strain rates, a honeycomb-like dislocation network develops within the BCC grains. Nanoscale dislocation cells are also observed, with a small number undergoing incomplete dynamic recrystallization to form ~100 nm defect-free recrystallized grains, as shown in Figure 9c and Figure 14b. Furthermore, a BCC→O′ phase transformation occurs within certain BCC grains, as illustrated in Figure 9(e_1_,e_2_). Concurrently, the dislocation density in the HCP phase increases and stacking faults becomes evident. Therefore, in addition to massive dislocation nucleation, slip, and interaction within the alloy, high-strain-rate deformation also induces incomplete dynamic recrystallization and phase transformation. The evolution of these deformation mechanisms under varying strain rates also plays an important role in the strain-rate-strengthening effect observed in the Ti61V5 alloy.

As shown in Figure 9c and Figure 11(d_1_–d_3_), after dynamic compression, nanoscale new grains were formed within some regions, indicating the occurrence of incomplete dynamic recrystallization. EBSD analysis was performed on the recrystallized shear band, and the results are shown in Figure 14b,c. The data reveal that the recrystallized grains exhibit no significant crystallographic texture, with an average grain size of approximately 345 nm. According to the grain boundary distribution, low-angle grain boundaries account for about 32%, while high-angle grain boundaries make up approximately 68%, indicating that most recrystallized grains are separated by well-defined boundaries. Considering the short deformation duration under a strain rate of 5020/s and during forced shear (e.g., approximately 70 μs for forced shear), diffusion-controlled dynamic recrystallization is unlikely to occur within this timeframe. Therefore, a rotational dynamic recrystallization mechanism (RDRX) is proposed to explain the recrystallization observed in Figure 9c and Figure 14b. According to the RDRX mechanism, under the influence of external stress and the driving force of interfacial energy minimization, fragmented low-angle subgrains rotate progressively to form high-angle grain boundaries (typically >30°), ultimately resulting in the formation of fully recrystallized grains [55]. The driving force for this dynamic recrystallization process is described by Equation (2) [56]:

(2) $$t=\frac{TL_{1}kf(\theta )}{4\delta \eta D_{b0}exp\left(-Q_{b}/RT\right)}$$

Here, t is the deformation time, T is the temperature, $L_{1}$ is the average diameter of the subgrains, k is the rate constant, δ is the grain boundary thickness, η is the grain boundary energy, $D_{b0}$ is the pre-exponential factor for grain boundary diffusion, and $Q_{b}$ is the activation energy for grain boundary diffusion, which is approximately 45% of the lattice diffusion activation energy Q. θ is the grain boundary rotation angle of the subgrains, which can be calculated using Equation (3):

(3) $$\begin{array}{c} f(\theta )=\frac{3tan\;\theta -2cos\;\theta }{3-6sin\;\theta }+\frac{2}{3}-\frac{4\sqrt{3}}{9}\ln\frac{2+\sqrt{3}}{2-\sqrt{3}}\\ +\frac{4\sqrt{3}}{9}\ln\frac{tan\left(\frac{\theta }{2}\right)-2-\sqrt{3}}{tan\left(\frac{\theta }{2}\right)-2+\sqrt{3}} \end{array}$$

For the Ti61V5 alloy, it is difficult to accurately determine the above parameters. However, they can be reasonably estimated using typical values for BCC-Ti alloys [57]: *δ* = 5.8 × 10^−10^ m, *η* = 1.19 J/m^2^, $D_{b0}$ = 2.8 × 10^−5^ m^2^/s, *Q* = 312 kJ/mol, *k* = 1.38 × 10^−23^ J/K, *R* = 8.314 J/(K·mol). As for the deformation temperature *T* within the alloy, it is assumed that dynamic compression occurs under quasi-adiabatic conditions, due to the generally low thermal conductivity of titanium alloys. Based on this assumption, the deformation-induced temperature rise Δ*T* can be estimated using Equation (4) [58]:

(4) $$\Delta T=\frac{0.95\eta }{\rho c}\int \sigma d\varepsilon$$

Here, *ρ* is the density of the Ti61V5 alloy, taken as 4.82 g/cm^3^; *c* is the specific heat capacity, for which the value of BCC-Ti is adopted as 0.52 J/(g·K) [57]; *σ* is the true stress during dynamic loading; and *ε* is the true strain. By substituting the above parameters into Equation (4), the temperature rise Δ*T* caused by deformation at a strain rate of 5020/s is estimated to be approximately 460 K. Considering that the shear band is a region of concentrated stress and temperature rise, it is inferred that the temperature increase within the recrystallized adiabatic shear band exceeds that observed after compression at 5020/s. Therefore, the temperature within the shear band is estimated to reach approximately 800 K (517 K + 293 K). Using this value in Equation (2), the rotation angle–time relationship for subgrain boundaries is calculated, as shown in Figure 14a. As illustrated in the figure, subgrains smaller than 300 nm can complete a boundary rotation of 30° within approximately 50 μs, while 500 nm subgrains can achieve a rotation of about 28° within 70 μs. These results demonstrate that the RDRX mechanism can reasonably explain the occurrence of recrystallization observed in the Ti61V5 alloy during high-speed dynamic compression.

### 4.3. Comparative Mechanical Properties and Application Prospects of Ti61V5 Alloy

**Figure 15:**
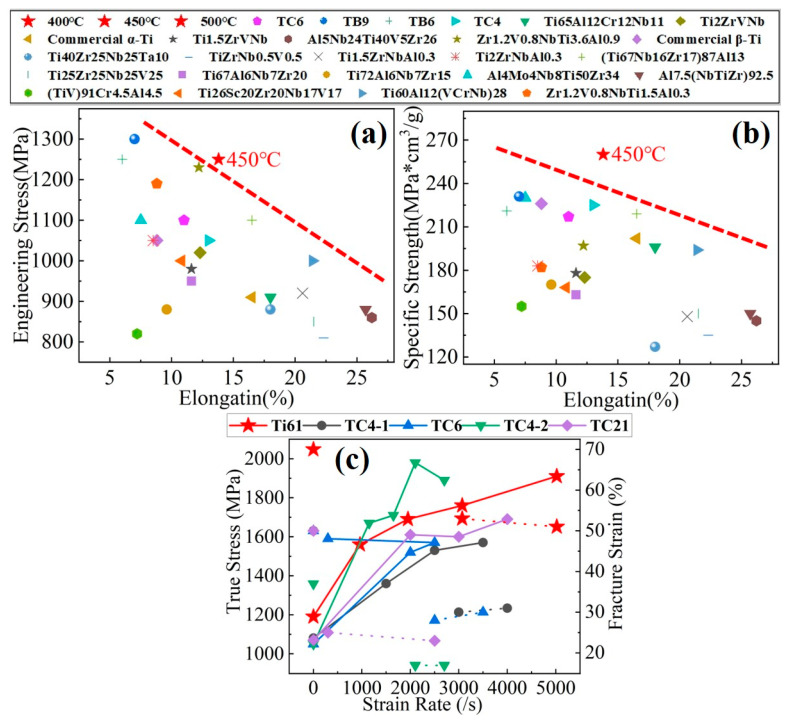
Comparison of the Ti_61_Al_16_Cr_10_Nb_8_V_5_ alloy with conventional titanium alloys and lightweight MPEAs: (**a**) strength–ductility comparison; (**b**) specific strength–ductility comparison; (**c**) Comparison of dynamic strength and fracture strain.

Based on mechanical testing and microstructural observations of the Ti61V5 alloy under both quasi-static and dynamic loading conditions, this study has gained a relatively comprehensive understanding of its fundamental mechanical properties and strengthening mechanisms. To further clarify the positioning of Ti61V5 and evaluate its potential application scenarios, its quasi-static and dynamic mechanical performance was compared with that of conventional titanium alloys [59,60,61] and other lightweight MPEAs [62,63,64,65,66,67,68,69,70,71,72,73,74]. The comparison results are shown in Figure 15. As illustrated in Figure 15a, the tensile strength of the Ti61V5 alloy is significantly higher than that of traditional titanium alloys such as TC4 and TC6, while also maintaining good ductility. Figure 15b shows that the specific strength of Ti61V5 reaches 262 MPa·cm^3^/g, which greatly exceeds that of TC4, TC6, and several lightweight MPEAs, highlighting its outstanding specific strength. As shown in Figure 15c [75,76,77,78,79], the Ti61V5 alloy exhibits a significant strain-rate-strengthening effect. With increasing strain rate, the alloy’s strength increases markedly and is significantly higher than that of conventional titanium alloys. Meanwhile, its fracture strain is also noticeably higher compared to traditional titanium alloys, demonstrating excellent dynamic compressive strength and fracture strain.

In summary, the Ti61V5 alloy exhibits a high tensile strength of 1260 MPa, an ultra-high specific strength of 262 MPa·cm^3^/g, a favorable elongation of 12.8%, and outstanding dynamic compressive mechanical properties. These characteristics indicate that the Ti61V5 alloy holds great potential for applications in military armor protection and aerospace structural components.

## 5. Conclusions

In this study, the quasi-static and dynamic mechanical properties of the Ti_61_Al_16_Cr_10_Nb_8_V_5_ alloy were systematically evaluated. The microstructural evolution and crack behavior under various deformation conditions were thoroughly characterized and analyzed. The main conclusions are as follows:

(1) The Ti61V5 alloy consists of an equiaxed BCC matrix with an average grain size of ~35 μm. Nano-sized B2 precipitates are uniformly dispersed within the matrix, and approximately 3.5% of the microstructure comprises an HCP phase. The measured density is ~4.82 g/cm^3^. Quasi-static mechanical testing reveals a yield strength of 1110 MPa, an ultimate tensile strength of 1260 MPa, and an elongation of 12.8%, corresponding to an ultra-high specific strength of ~262 MPa·cm^3^/g. Dynamic compression tests indicate a pronounced strain-rate-strengthening effect, with the alloy achieving a flow stress of 1850 MPa and a compressive strain of 50% at a strain rate of 5020/s. These results suggest that the Ti61V5 alloy possesses high strength, exceptional specific strength, and good ductility, offering significant potential for applications in military armor and aerospace structures.
(2) At low strain rates (0.001/s–10/s), the strain rate sensitivity coefficient (m) is approximately 0.0088, and plastic deformation is dominated by dislocation slip. The alloy initially activates slip bands along the close-packed {011} planes. Cross-slip interactions between slip bands lead to the formation of jogs, dislocation loops, and forest dislocations. With increasing strain, slip along the {112} planes is also activated. These slip systems bypass obstacles such as precipitates and dislocation structures through cross-slip and climb, resulting in a high density of mixed dislocations predominantly governed by screw components. Moreover, the B2 phase contributes to strengthening via dislocation shearing mechanisms, while the HCP phase strengthens the matrix through a dislocation bypass mechanism.
(3) At high strain rates (960/s–5020/s), the strain rate sensitivity coefficient increases significantly to ~0.0985, accompanied by intense dislocation interactions. In addition to {011} and {112} planes, the alloy also activates high-index slip systems {123} under the combined effects of high temperature and stress. This leads to a substantial increase in jogs and the forest dislocation density, which effectively impedes dislocation motion and enhances strength. A notable increase in the fraction of edge dislocation-dominated mixed dislocations is also observed, indicating that more dislocations bypass obstacles through climb and cross-slip, thereby improving plastic deformability. Additionally, localized BCC→O′ martensitic transformations occur in some grains, and the dislocation and stacking fault density within the HCP precipitates increases significantly.
(4) Forced shear testing reveals the formation of shear bands with widths ranging from 10 to 20 μm, which can be categorized into two types. At lower strains, plastic-flow shear bands dominate, within which grains undergo severe plastic deformation and exhibit amorphous-like structures. At higher strains, a small number of recrystallized shear bands are observed, characterized by the formation of new equiaxed grains with an average size of ~345 nm. This recrystallization behavior is explained well by the mechanism of rotational dynamic recrystallization. Microcracks and microvoids initiate at various locations within the shear bands and gradually expand with increasing strain. Crack propagation may terminate when intersecting with microvoids outside the shear band, whereas interaction with internal microcracks can cause slight deflection followed by continued propagation.

## Figures and Tables

**Table 1:** Compressive stress statistics of the Ti_61_Al_16_Cr_10_Nb_8_V_5_ alloy at various strain rates.

True Stress (MPa)	0.001/s	0.1/s	10/s	960/s	1950/s	3070/s	5020/s
Yield Stress	1190	1210	1230	1470	1630	1670	1810
Ultimate Stress	1270	1280	1350	1560	1690	1760	1910
Flow Stress	1290	1310	1330	1460	1550	1630	1810

**Table 2:** Dislocation extinction table of Ti_61_Al_16_Cr_10_Nb_8_V_5_ alloy under incident electron beam B = [-111] with different g vectors.

g/b	±1/2[111]	±1/2[-111]	±1/2[1-11]	±1/2[1-11]
(110)	√	×	×	√
(101)	√	×	√	×
(01-1)	×	×	√	√

**Table 3:** Statistical results of dislocation types in Ti_61_Al_16_Cr_10_Nb_8_V_5_ alloy at 50% compressive strain.

Dislocation No.	Burgers Vector (b)	Dislocation Line Direction (u)	Dislocation Character
1 (Dislocation Array)	±1/2[11-1]	[1,15,19]	Mixed-Edge dominated
2 (Dislocation Array)	±1/2[1-11]	[01-2]	Mixed-Screw dominated
3 (Dislocation Array)	±1/2[11-1]	[01-2]	Mixed-Screw dominated
4	±1/2[1-11]	[-132]	Edge
5	±1/2[1-11]	[1-11]	Mixed-Screw dominated
6 (Dislocation Array)	±1/2[111]	[-3,19,15]	Mixed-Screw dominated
7	±1/2[111]	[012]	Mixed-Screw dominated
8	±1/2[1-11]	[-12-1]	Mixed-Screw dominated
9 (Dislocation Array)	±1/2[11-1]	[01-2]	Mixed-Screw dominated

**Table 4:** Statistical results of dislocation types in the Ti_61_Al_16_Cr_10_Nb_8_V_5_ alloy under a strain rate of 5020/s.

Dislocation No.	Burgers Vector (b)	Dislocation Line Direction (u)	Dislocation Character
1	±1/2[111]	[2-3-1]	Mixed-Edge dominated
2 (Dislocation Array)	±1/2[1-11]	[2-3-1]	Mixed-Edge dominated
3 (Dislocation Array)	±1/2[11-1]	[021]	Mixed-Edge dominated
4 (Dislocation Array)	±1/2[1-11]	[021]	Mixed-Edge dominated
5	±1/2[1-11]	[021]	Mixed-Edge dominated
6 (Dislocation Array)	±1/2[1-11]	[021]	Mixed-Edge dominated
7	±1/2[1-11]	[011]	Mixed-Edge dominated
8	±1/2[111]	[1,15,11]	Mixed-Edge dominated
9 (Dislocation Array)	±1/2[11-1]	[021]	Mixed-Screw dominated

## Data Availability

The raw data supporting the conclusions of this article will be made available by the authors on request.

## Supplementary Materials

The following supporting information can be downloaded at: https://www.mdpi.com/article/10.3390/ma18143245/s1, Figure S1. Dislocation morphologies and extinction contours of the Ti61V5 alloy after quasi-static compression at the same location as in Figure 6 of the manuscript, observed under different *g* vectors with the incident electron beam along B = [011]. Figure S2. Dislocation morphologies and extinction contours of the Ti61V5 alloy after dynamic compression, observed at the same location as in Figure 10 of the manuscript under different *g* vectors with the incident electron beam along B = [011]. Figure S3. Dark-field TEM images of the B2 phase morphology in the Ti61V5 alloy and its size statistics.
