# Supplementary material for: Ultra-High Strength and Specific Strength in Ti61Al16Cr10Nb8V5 Multi-Principal Element Alloy: Quasi-Static and Dynamic Deformation and Fracture Mechanisms
Source: Materials (Basel). 2025 Jul 10;18(14):3245. doi: 10.3390/ma18143245 (PMC12298045; doi:10.3390/ma18143245)
Supplement: Supplementary file 1 [file materials-18-03245-s001.zip › materials-3727624-supplementary.pdf]

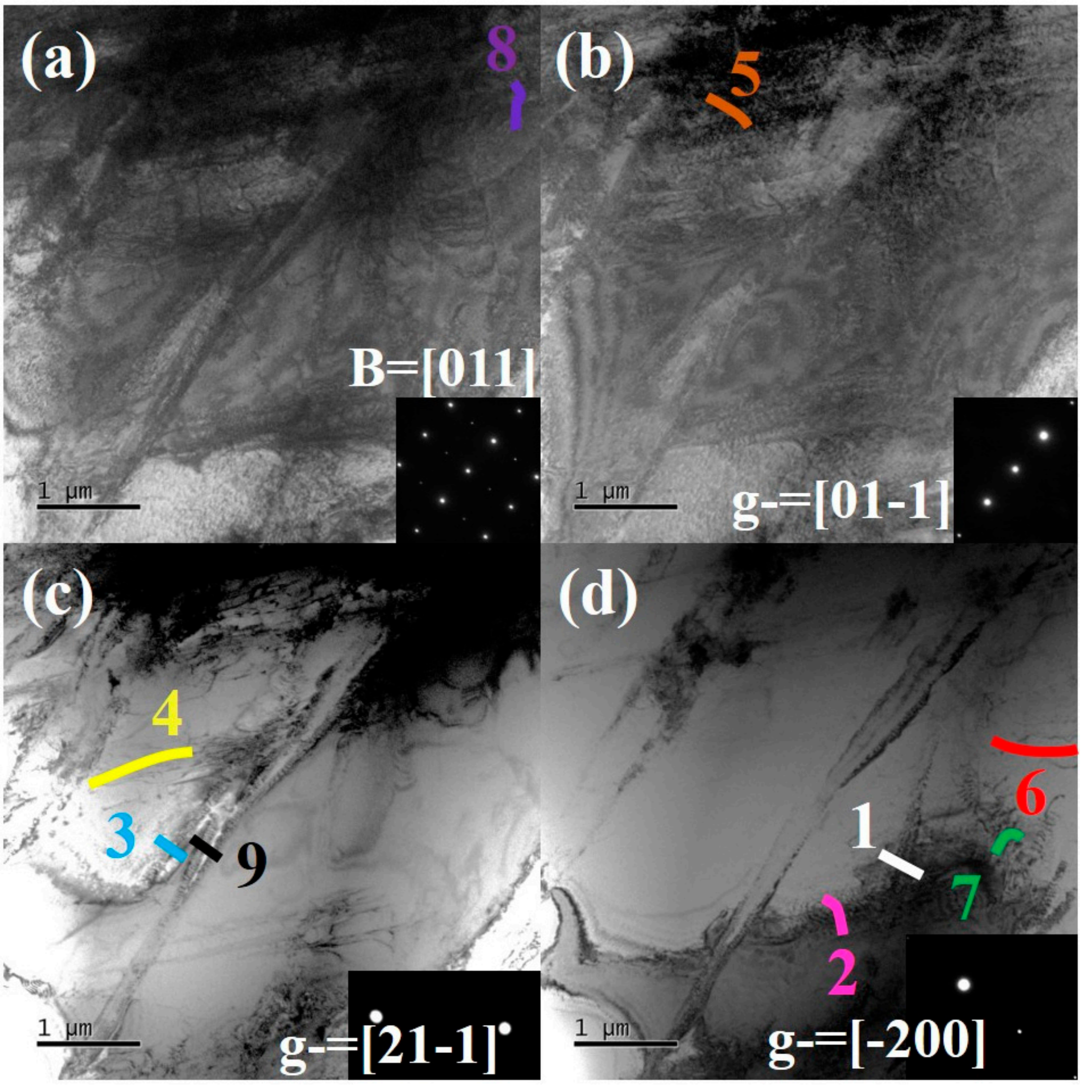

Figure S1. Dislocation morphologies and extinction contours of the Ti61V5 alloy after quasi-static compression at the same location as in Figure 6 of the manuscript, observed under different *g* vectors with the incident electron beam along B = [011].

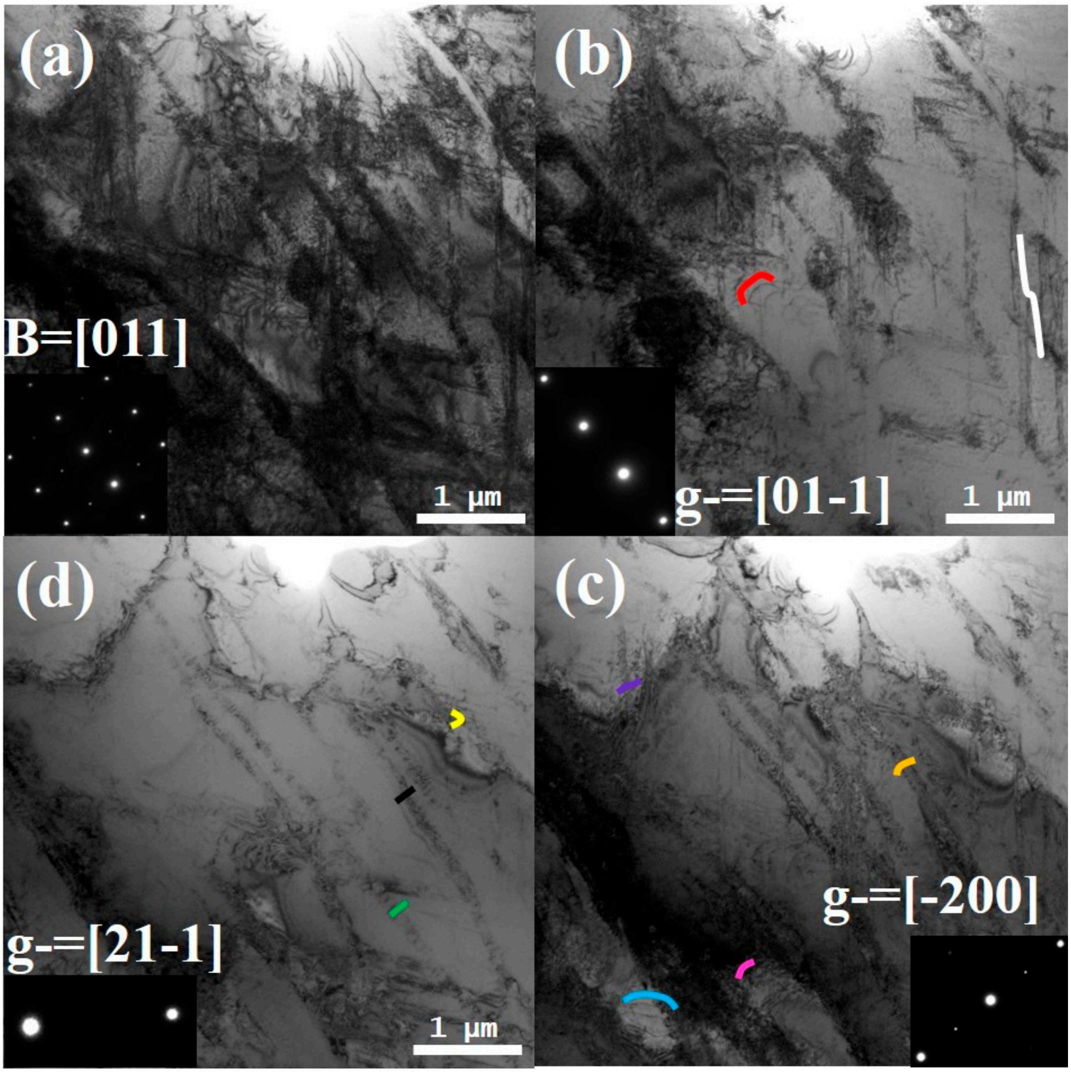

Figure S2. Dislocation morphologies and extinction contours of the Ti61V5 alloy after dynamic compression, observed at the same location as in Figure 10 of the manuscript under different *g* vectors with the incident electron beam along B = [011].

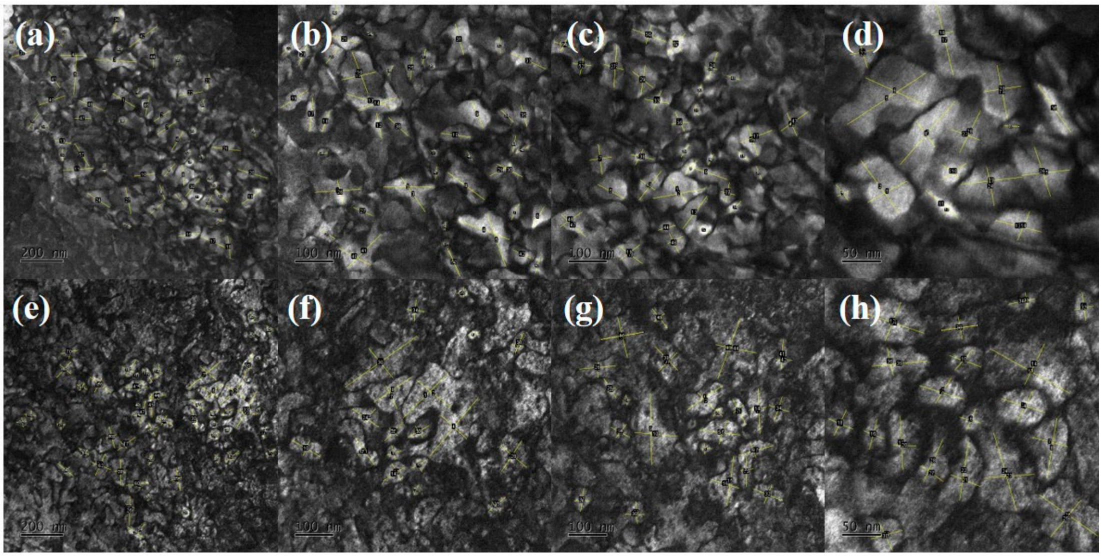

Figure S3. Dark-field TEM images of the B2 phase morphology in the Ti61V5 alloy and its size statistics.
